# Change in University Student Health Behaviours after the Onset of the COVID-19 Pandemic

**DOI:** 10.3390/ijerph20010539

**Published:** 2022-12-29

**Authors:** Alex Patin, Joel Ladner, Marie-Pierre Tavolacci

**Affiliations:** 1 Centre d’Investigation Clinique-Centre de Ressources Biologiques (CIC-CRB 1404), CHU ROUEN, 76000 Rouen, France; 2 Department of Epidemiology and Health Promotion, CHU ROUEN, 76000 Rouen, France; 3 INSERM 1073, Univ Rouen, 76000 Rouen, France

**Keywords:** COVID-19, binge drinking, cannabis, tobacco, physical activity, university students

## Abstract

Introduction: The COVID-19 pandemic has caused many disruptions in the lives of the population. In particular, the health behaviours of university students were impacted. Thus, the aim of this study was to evaluate the evolution of these behaviours from before the COVID-19 period to May 2021, during which lockdowns or curfews were in effect. Methods: Two retrospective online cross-sectional studies were conducted among university students in Normandy in May 2020 and May 2021. Socio-demographics and academic characteristics were collected. Tobacco smoking, binge drinking, cannabis use, and moderate and vigorous physical activity were collected for the 4 weeks before the COVID-19 lockdown in May 2020 and in May 2021. Results: Overall, 6991 university students were included in the study (3483 in 2020 and 3508 in 2021) with a mean age of 20.8 (standard deviation = 2.5) and 73.4% of women. After logistic regression, binge drinking (occasional and regular), cannabis use (occasional), moderate physical activity (regular), and vigorous physical activity (occasional) decreased in 2020 and 2021 compared to the pre-COVID-19 period. Tobacco smoking (occasional) and vigorous physical activity (regular) decreased only in 2020. Regular tobacco and cannabis use did not change significantly in 2020 and 2021 compared to the pre-COVID-19 period. Discussion: Student health behaviours changed in May 2020 and May 2021 due to the implementation of measures restricting mobility and social interaction. Even if some risky consumption decreased in 2020 after the first lockdown, there was no rebound phenomenon in 2021: consumption either remained lower or similar to the pre-COVID-19 period. These behaviours need to be monitored in the future to assess the long-term effects of these restrictions on student health behaviours.

## 1. Introduction

The World Health Organization (WHO) declared the coronavirus disease (COVID-19) outbreak a public health emergency of international concern and categorized it as a pandemic in March of 2020 [1]. With the aim of limiting virus transmission, many countries adopted exceptional measures restricting mobility and social interaction. In France, the first lockdown took place from 17 March to 10 May 2020 [2]. Universities were immediately closed, which led to immediate changes in teaching and examinations, including the introduction of distance learning courses. Since the end of this first lockdown, new measures have been taken by the government according to the evolution of the epidemic, including the partial continuation of university courses utilising distance learning and a curfew during the 2020–2021 university year. For students, the COVID-19 pandemic involved rapid changes in their personal lives, as well as their student life, and uncertainty in whether they could complete their academic year. In addition, due to social distancing measures, many students moved back to their parents’ homes or lived in more isolated conditions in their student homes. These changes were likely to impact their mental health and well-being [3]. The COVID-19 pandemic has had a strong negative impact on the mental health and well-being of students, including more anxiety, stress, anger, fear, and depressive symptoms [4].

During the COVID-19 lockdown, people self-reported drinking slightly more alcohol, and the number of cigarettes smoked per day only marginally increased compared to before the lockdown. Among young adults, studies showed a decreasing trend in binge drinking, cannabis use, and tobacco smoking during the first COVID-19 lockdown [5,6,7] or even no change during the COVID-19 pandemic [8]. Unfavourable changes for alcohol consumption and smoking since the onset of the COVID-19 pandemic was associated with higher depression, anxiety, and stress symptoms [7,9]. These lockdowns heavily also limited people’s physical activity opportunities [10] with an increase in both physical activity and sitting time [11,12]. This limitation of physical activity was a concern, and experts argued in favour of continued exercise to avoid severe physical problems caused during COVID-19 [13].

It was recommended to continue to monitor the evolution of students’ behaviour during the COVID-19 pandemic [14]. The sudden lockdown, followed by restrictions on social contact, mobility, and face-to-face classes at the university, may have resulted in different health behaviours throughout the year after the pandemic onset. The COVID-19 pandemic may have increased the vulnerability that accompanies the development and establishment of risky health behaviour during early adulthood. Substance use could have been exacerbated following restrictions imposed by the first lockdown, and it was possible that expected that physical activity could return to the pre-COVID-19 level. Thus, the aim of this study was to evaluate the evolution of the health behaviours after the first lockdown and one year later compared to the pre-COVID-19 pandemic period among university students.

## 2. Methods

### 2.1. Study Design

Two repeated online cross-sectional studies at a French university were conducted in May 2020 [7] in the last weeks of the first lockdown and one year later in May 2021. University-wide email distribution lists were used to invite students to participate in these two studies.

### 2.2. Data Collection

#### 2.2.1. Socio-Demographic Characteristics

Data were collected on gender, type of academic course (law, literature, healthcare, and sciences), and academic year of study, which was further categorized into the following: year 1, years 2 and 3, years 4 and 5, and year 6+. Students also reported data on their living accommodations.

#### 2.2.2. Mental Health

Depression was assessed using the eight items of the Centre for Epidemiologic Studies—Depression (CES-D8) [15] scale and has shown adequate psychometric properties (Cronbach’s alpha of 0.82). The response values are scored on a 4-point Likert scale (range 0 to 3) and on the CESD-8 scale from 0 to 24, with higher scores indicating a higher frequency of depressive complaints. Academic stress was assessed using a Likert scale with five answers from totally agree to totally disagree for three different categories: stress regarding changes in teaching methods, increased academic workload, and concern about not completing the academic year.

#### 2.2.3. COVID-19 Infection

Students were asked whether they had been infected with COVID-19 (validated by test or by health care provider). Their concern regarding COVID-19 was assessed on a scale from 0 to 10, based on two categories: worried about becoming severely ill from a COVID-19 infection and worried about a relative becoming severely ill from a COVID-19 infection.

#### 2.2.4. Health Behaviours

To assess behaviours before the COVID-19 pandemic, questions were preceded with the phrase ‘during the month before the COVID-19 measures’. To assess behaviours in May 2020 and May 2021, questions were preceded with the phrase ‘during the last week’. Students reported their frequencies of the following: tobacco smoking (including cigarettes, cigars, or e-cigarettes), binge drinking (defined as six or more glasses of alcohol on a single occasion), and cannabis use. Frequencies of binge drinking were categorized as never; occasional, when the behaviour occurred at least once a week; and regular, when the behaviour occurred more than once a week. Students reported their frequencies of moderate physical activity, including cycling or walking for at least 30 min, and vigorous physical activity, including lifting heavy weights, running, aerobics, or fast cycling for at least 30 min. Frequencies were categorized as never; occasional, when the student practiced at least once a week; and regular, when the student practiced more than once a week.

### 2.3. Statistical Analysis

Participants aged 18 to 30 years who completed questionnaire on health behaviours were included. The sample size was estimated as at least 10% of Rouen-Normandy University’s student population (30,000) at each study. A variable ‘period’ was created with three modalities: before COVID-19, May 2020, and May 2021. The ‘pre-COVID-19’ modality aggregated students’ responses on their health behaviours prior to the COVID-19 pandemic from questionnaires distributed in 2020 and 2021.

Continuous variables were expressed as mean with standard deviation (SD) and compared with Student’s *t*-tests. Qualitative variables were reported as percentages and compared with a chi-squared test. A multivariate logistic regression model was fitted to assess the evolution of each health behaviour with the pre-COVID-19 period as a reference. Age, gender, curriculum, and year of study were the adjusted variables. For each health behaviour, the adjusted odds ratios (AOR) were provided with their 95% confidence interval (CI). All statistical tests were two-tailed, and a p-value of less than 0.05 was considered statistically significant.

All statistical analysis was performed using R—a language and environment for statistical computing (R Foundation for Statistical Computing, Vienna, Austria).

## 3. Results

The intention to sample at least 10% of students was achieved with 3483 university students being included in the first study (participation rate of 11.4%) and 3508 university students being included in the second study (participation rate of 11.0%). Among the 6991 students, 73.4% were women and the mean age was 20.8 (SD = 2.5). Concerning the curricula, 33.9% of students were in healthcare, 28.2% in literature, 22.2% in sciences, and 15.7% in law. Living accommodation, COVID-19 infection, CESD-8 score, academic stress, and COVID-19 concern for a loved one are displayed in Table 1.

**Figure 1:**
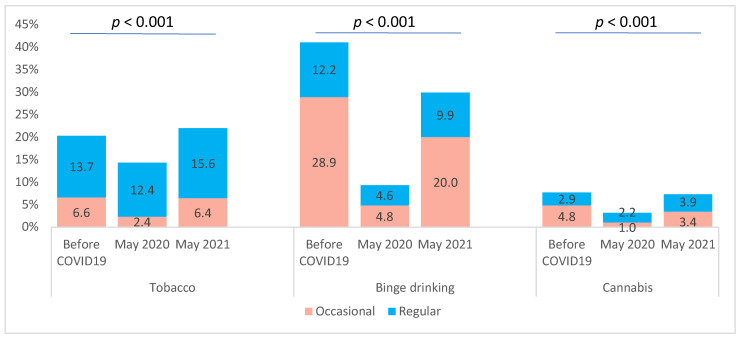
Tobacco smoking, binge drinking, and cannabis use of university students before the COVID-19 pandemic, in May 2020 and May 2021.

**Figure 2:**
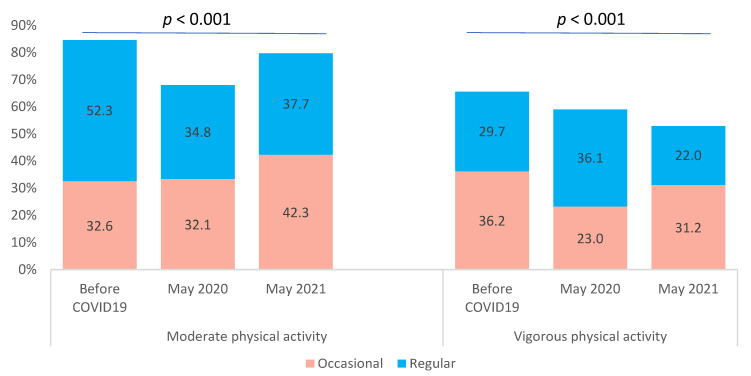
Moderate and vigorous physical activity of university students before COVID-19 pandemic, in May 2020 and May 2021.

A decrease in tobacco smoking, binge drinking, and cannabis use was observed between the pre-COVID-19 period and May 2020. In May 2021, these behaviours increased and seemed to almost reach the levels of the pre-COVID-19 period (Figure 1). Similar results were observed for moderate physical activity. In contrast, vigorous physical activity decreased from the pre-COVID-19 period to May 2021 (Figure 2).

**Figure 3:**
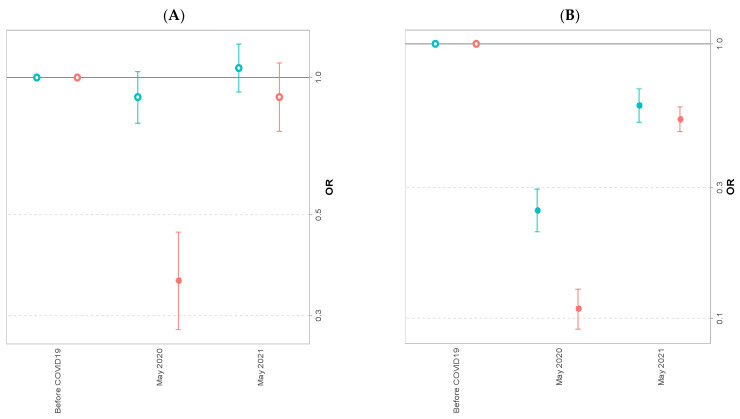

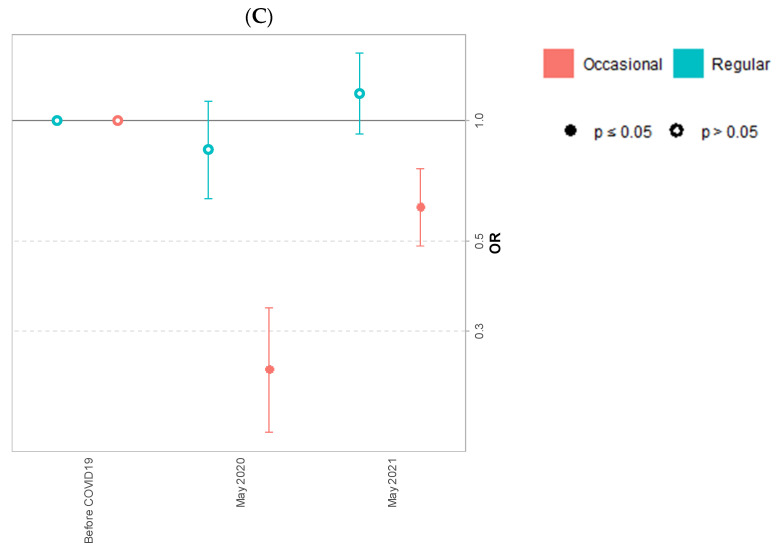
Adjusted odds ratio and Confidence Interval 95% for occasional and regular tobacco smoking (**A**), binge drinking (**B**) and cannabis use (**C**) of university students in May 2020 and May 2021 compared to the pre-COVID-19 period.

After logistic regression, regular smoking was similar in May 2020 and May 2021 to the pre-COVID-19 period, occasional smoking in May 2021 was similar to the pre-COVID-19 period after having decreased in May 2020 (AOR) = 0.36 95% CI [0.28, 0.46]) (Figure 3A). Occasional and regular binge drinking decreased in May 2020 and May 2021 compared to the pre-COVID-19 period (in 2020: AOR = 0.11 95% CI [0.09, 0.13] for occasional and AOR = 0.24 95% CI [0.20, 0.29] for regular; in 2021: AOR = 0.53 I95% CI [0.48, 0.59] for occasional and AOR = 0.60 95% CI [0.52, 0.70] for regular) (Figure 3B). Regular use of cannabis did not change in May 2020 and May 2021; however, occasional cannabis use decreased in 2020 and 2021 compared to the pre-COVID-19 period (AOR = 0.24 95% CI [0.17, 0.34] and AOR = 0.61 95% CI [0.49, 0.76], respectively) (Figure 3C).

**Figure 4:**
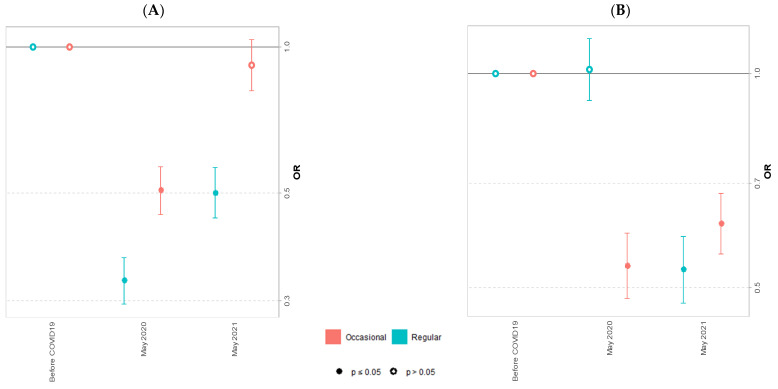
Adjusted odds ratio and Confidence Interval 95%for occasional and regular moderate (**A**) and vigorous (**B**) physical activity of university students in May 2020 and May 2021 compared to the pre-COVID period.

Moderate regular physical activity decreased in May 2020 and May 2021 (AOR = 0.33 IC95% [0.29, 0.37] and AOR = 0.50 IC95% [0.44, 0.56], respectively). Occasional moderate physical activity decreased in May 2020 (AOR = 0.51 IC95% [0.45, 0.57]) but returned to the levels of the pre-COVID-19 period in May 2021 (Figure 4A). Regular vigorous activity did not change in May 2020 but decreased in May 2021 (AOR = 0.53 IC95% [0.48, 0.59]). Occasional vigorous activity decreased in May 2020 and May 2021 (AOR = 0.54 IC95% [0.48, 0.60] and AOR = 0.61 IC95% [0.56, 0.68], respectively) (Figure 4B).

## 4. Discussion

The main findings of this study highlighted changes in French university students’ health behaviours in May 2020 and May 2021 as national measures were implemented with restriction of mobility and social interactions to limit COVID-19 transmission. Overall, the risk of substance use was not higher than before the onset of the COVID-19 pandemic. By May 2021, regular smoking had returned to pre-COVID-19 levels, while occasional smoking remained lower. Binge drinking had significantly decreased in May 2020 and May 2021, although there seems to have been a trend towards a return to pre-COVID-19 period because parties were possible even though the university was physically closed and using distance learning. During early adulthood, socialization with peers provides and facilitates opportunities for consumption; therefore, the constraint of social isolation due to COVID-19 made it more difficult to access risky substances [16,17] In the general population, studies reported that COVID-19 measures led to increased tobacco smoking and being a student was a protective factor for increased tobacco smoking [18].

The decreases in occasional tobacco smoking, binge drinking, and occasional cannabis use in 2020 (total lockdown) can be explained by the fact that these are activities often undertaken in groups, especially among students during parties, which was limited by the rules put in place by the government, whereas groups of small numbers were possible in 2021 (curfews and partial lockdown) [19].

On the other hand, Jackson showed an increase in the frequency of alcohol consumption but a decrease in the quantity of alcohol consumption related to the COVID-19 pandemic [20]. Social isolation had a negative impact on the psychological and mental health of the students (academic stress and worrying about COVID-19 infection), which may have resulted in this increase in the frequency of alcohol consumption [21]. Moreover, some adolescents may have initiated substance use to cope with the psychological discomfort and negative feelings associated with the COVID-19 situation. [22]. Regular cannabis use did not change, while occasional cannabis use significantly decreased. The changes in student health behaviour in May 2020 shown this study are congruent with the literature [23,24]. Restrictions on mobility and social interactions could have increased the obstacles for the illicit trade of cannabis, thus reducing its availability and accessibility [17,22].

Despite recommendations that home lockdown should not hinder people from being physically active [25], occasional moderate physical activity significantly decreased in May 2020 but increased when students were no longer confined and were able to walk to university, internships, or jobs. Regular vigorous physical activity did not decrease in 2020, perhaps because the first sudden confinement ended leisure activities but allowed regular vigorous physical activities at home to cope with stress [26]. In 2021, fitness centres and team sports had not fully recovered and restarting daily activities could explain the decrease of the regular vigorous activity. In Spain, the student population and highly active men experienced a decrease in self-reported daily physical activity and increased sedentary time during COVID-19 isolation. In Germany, the authors found that sports activity declined, whereas recreational screen time increased. 

The principal limitations of this study were the convenience sample which caused a possible selection bias; however, the proportion of women in our sample was similar to the population of university students in Rouen [27]. Health behaviour was assessed through retrospective self-reporting measures, which may have led to memory bias; however, the anonymous nature of the questionnaire limited desirability bias. Finally, in accordance with the literature, our study population was predominantly female. The main strength of this study is the two-fold measurement of health behaviours at different time points, after the first lockdown and one year later after the onset of the COVID-19 pandemic, among students in the same university. Moreover, the statistical power, especially with the size of the sample, represents a strength.

## 5. Conclusions

This study was conducted in a large population of university students in France, focusing on health-related behaviours (tobacco smoking, binge drinking, cannabis use, and physical activity) after the onset of COVID-19 pandemic. Even though a certain amount risky consumption decreased in 2020 after the first lockdown, there was no rebound phenomenon in 2021. The need for freedom, social contact, and entertainment is particularly prominent in adulthood, including university students, which made social restrictions particularly difficult for mental health [28]. Coping mechanisms during the first lockdown and the social motivations the following year did not lead to an increase in risky behaviours as might have been feared [29]. Physical activity decreased after the onset of the COVID-19 pandemic without returning to pre-pandemic levels. It would be interesting to record the body mass index of the students, which may have increased due to the decline in physical activity.

When, in the near future, COVID-19 is no longer a global threat to public health due to mass population vaccination and there are no remaining restrictions, further studies will be needed to evaluate whether university students’ health behaviours returned to baseline levels or remained lower than initially measured

Health-promotion strategies directed at adopting or maintaining positive mental health should be developed for university students to better manage future lockdown periods. As suggested by Bao, improving e-health literacy and self-efficacy could improve college students’ healthy lifestyle behaviours [30]. Recommendations to maintain health during the ongoing COVID-19 pandemic that specifically target university student populations are needed.

## Figures and Tables

**Table 1:** Characteristics of the university students (Study May 2020 *n* = 3483; study May 2021 *n* = 3508).

Characteristics	Study May 2020 (*n* = 3483)		Study May 2021 (*n* = 3508)		*p*
Living accommodation: with parents, *n* (%)	2418	(69.4)	1788	(51.0)	<0.001
COVID-19 infection, *n* (%)	274	(7.9)	648	(18.5)	<0.001
CESD-8 scale, mean (SD)	8.6	(5.06)	11.1	(5.94)	<0.001
Stressed with changes in teaching methods, *n* (%)					<0.001
Totally agree and agree	2488	(71.4)	2479	(70.8)	
Do not agree, do not disagree	643	(18.5)	513	(14.6)	
Totally disagree and disagree	352	(10.1)	512	(14.6)	
Increased academic workload, *n* (%)					<0.001
Totally agree and agree	2030	(58.3)	1998	(57.0)	
Do not agree, do not disagree	962	(27.6)	973	(27.8)	
Totally disagree and disagree	491	(14.1)	533	(15.2)	
Worried about not completing the academic year, *n* (%)					<0.001
Totally agree and agree	2240	(64.3)	1773	(50.6)	
Do not agree, do not disagree	735	(21.1)	596	(17.0)	
Totally disagree and disagree	508	(14.6)	1135	(32.4)	
Worry COVID-19 infection, mean (SD)	3.6	(3.31)	3.6	(3.26)	0.97
COVID-19 infection concern for a loved one, mean (SD)	7.0	(2.97)	7.8	(2.66)	<0.001

## Data Availability

Data are available on request.
